# Frailty in Older Adults and Internal and Forced Migration in Urban Neighborhood Contexts in Colombia

**DOI:** 10.3389/ijph.2023.1605379

**Published:** 2023-05-05

**Authors:** Herney Rengifo-Reina, Tonatiuh Barrientos-Gutiérrez, Nancy López-Olmedo, Brisa N. Sánchez, Ana V. Diez Roux

**Affiliations:** ^1^ Center for Population Health Research, National Institute of Public Health (Mexico), Cuernavaca, Mexico; ^2^ Department of Epidemiology and Biostatistics, Drexel University, Philadelphia, PA, United States; ^3^ Urban Health Collaborative, Dornsife School of Public Health, Drexel University, Philadelphia, PA, United States

**Keywords:** Colombia, older adults, urban areas, migration, frailty, neighborhoods, host population

## Abstract

**Objective:** We investigated the association between the density of internal human migration, in the urban neighborhood, on frailty in the older adult population in Colombia.

**Methods:** The data used in this study are from four Colombian population surveys. We analyzed 633 census tracts with a sample of 2,194 adults 60 years and over for frailty (measured using the Fried criteria). We considered the proportion of inhabitants in a census tract with a history of internal migration as the exposure variable considering three temporalities. For contextual forced migration, we identified two types: 5-year, and 1-year. Poisson multivariable regression models with two hierarchical levels (individual and census tracts) were estimated.

**Results:** The prevalence of pre-fragile/frailty was 80.63% [CI 95%: 77.67, 83.28]. The prevalence ratio were significantly higher for the older adults who live in neighborhoods where a higher proportion of internal migrants reside.

**Conclusion:** We conclude that older adults who lived in neighborhoods with a high proportion of internal migrants experience more frailty. Potential explanations are that neighborhoods with high internal migration could experience social (l increase in cultural heterogeneity, in the perception of insecurity, violence and physical conditions (pressure on local economies and services, leading elderly residents to compete for neighborhood resources), translated into social stress.

## Introduction

Latin American countries are experiencing an accelerated population aging process. The percentage of older adults in Latin America increased from 6% in 1965 to 11.8% in 2017 and is expected to reach 20% by 2050 (1). Colombia is in a moderately advanced aging stage, with 13.5% of its population over 60 years of age in 2020 (2); yet, its demographic transition has been fast, since in 1950 only 5% of the population had more than 60 years of age and by 2050 this percentage will be more than 23%. The acceleration of aging already poses a significant public policy and public health challenge for the country.

The elderly in Colombia are expected to experience an increase in the prevalence of non-communicable, degenerative and disabling diseases (3). Frailty is one of the most important syndromes in this age group, yet, little is known about how frailty interacts with social processes. Frailty is a biological syndrome by which biological reserve and resistance are diminished by stressors as a result of multiple physiological systems that become unbalanced (4, 5), leading to functional deterioration and adverse events that worsen health, decrease quality of life and can even cause death (6). The origin of frailty is multicausal (4, 6); however, the evidence has mainly focused on the magnitude and coexistence of individual and social care factors (7, 8) rather than contextual and neighborhood factors, such as human migration.

Colombia has experienced an important increase in internal migration, defined as a situation where a person moves within a country, outside of their usual residence (9), often related to forced migration due to conflict. More than 30% of the population in Colombia has relocated within the country in the past 3 decades (10), a third of them due to forced migration but also because of economic reasons and natural disasters (11). Human migration is a social determinant of health that can positively or negatively influence people’s health (12). Internal migration can affect an individual’s health through different pathways, including social stress and social cohesion (13).

Most of the evidence about the association between general migration and health/disease is observed in high-income countries (HICs) (14). Human migration has also been studied in African countries in the context of refugees, but most analyses have focused on the impacts on the host population around economic issues, mainly followed by the provision of public services and health, environment and infectious diseases (15). Nevertheless, it is unclear how this link is presented in Latin American Countries (LACs). Moreover, most of the studies have focused on assessing the effects of migration on migrants themselves’. However, little is known about whether contextual migration potentially impacts the host population (16–18), especially when considering older adults. In summary, the association between contextual migration as a social exposure and frailty among the elderly population in urban settings has been no been studied, as the existing literature revolves around the experience of the elderly as an international migrant, with a greater probability of frailty being observed in migrants from low-income countries (19, 20).

The large internal migration experienced by Colombia provides a unique opportunity to assess how the proportion of migration in a neighborhood influences frailty beyond the individual experience of migration. We aimed to assess the association between the proportion of people in a neighborhood who migrated within the country for forced and non-forced reasons and the prevalence of frailty in older adults in urban areas of Colombia.

## Methods

### Study Population

This is a cross-sectional study based on an analysis of secondary data. The data was obtained from four Colombian population surveys: Health, Wellbeing, and Aging (SABE—2015), Demography and Health (ENDS 2015), Nutritional Situation of Colombia (ENSIN 2015), and Mental Health (ENSM 2015), with a response rate of 77%, 93.4%, 85.0%, and 97.4%, respectively. The protocols and sample sizes for each survey are published elsewhere (3, 21–23). The sample design of each survey followed the guidelines of the master sample of households for health studies that was designed by the Ministry of Health and Social Protection (24). Groups of contiguous blocks of the same sector were formed and randomly sampled for each survey; thus, while surveys did not cover the same households they sampled the same census tracts. This creates an opportunity for linkage of contextual data across surveys. Based on this premise, census tracts with information on the four surveys (*n* = 633) were selected to represent urban neighborhoods, with a sample of 2,194 participants 60 years of age and older. This study was approved by the ethics, research, and biosecurity committee of the National Institute of Public Health of Mexico (Protocol 1183).

### Outcome Variables—Frailty

We obtained information on frailty from the SABE survey (3). We defined frailty using the criteria established by Fried et al. (5): 1) Self-reported unintended weight loss in the last 3 months; 2) Measured grip strength adjusted according to gender and body mass index; 3) Self-reported physical fatigue or exhaustion; 4) Observed difficulty walking; 5) Self-reported physical activity. Participants were considered frail or pre-frail if they experienced any of the five criteria.

### Individual-Level Covariates

We included individual-level social, demographic, and economic characteristics of the older adults from the SABE survey. We considered: sex, age in 5-year groups and educational level into four categories (neither, primary schooling, secondary schooling, university education). Marital status was categorized as living alone or with a partner (married, free union). Health insurance was categorized into insured (contributory regimen - formal workers and subsidized regimen—non-workers, subsidized by the government), or uninsured. We also identified if participants received a pension (yes/no). Socioeconomic position (SEP) was categorized into low, medium, and high according to the DANE classification (25). Living arrangements (with whom the participant lived) included living with a son/daughter and living with friends or caregivers. And the individual experience of migration (lifetime migrant).

### Exposure Variables—Contextual Internal Migration at the Census Tracts Level

Contextual migration information was obtained from the sociodemographic questionnaires of the four surveys. We considered internal migration as the change in residence within the same country for Colombian-born participants. We identified three types of contextual internal migration: 1) lifetime: participants that resided in a place other than the one where they were born and have remained in the current residence for the past 5 years; 2) 5-year: change of residence during the previous 5 years, and 3) 1-year: change of residence during the last 12 months. For contextual forced migration (main reason for changing residence was armed conflict or insecurity), we identified two types: forced displacement during the previous 5 years and forced displacement during the last 12 months.

We formed categories of total migration based on the distribution by quintiles for each of the temporal frames (where neighborhoods with less than 1% of migrants in their structure were in the first category): lifetime, 5 years and 1 year: <1%, 1%–2%, 3%–5%, 6%–10%, and 11% or more. For people with forced migration within 5 and 1 years, the categories were: <1%, 1%–2%, and 3% and more.

### Census Tract Level Covariates

At the census tract level, we included socioeconomic level (level 1: very-low to 6: high), and age distribution (proportion of people 0 to 14, 15 to 64, and 65 and over), which were obtained from the 2018 national census (26).

Although it is true that migration matrices are a fundamental tool for migration analysis, they are built from censuses and are given mainly for large administrative units of the countries (provinces and states). When performing analysis in smaller administrative units such as neighborhoods, the matrix becomes more complex due to the number of crossings and the difficulty of obtaining information at that level.

For this study, the migration matrix could not be fully structured because the information available was obtained only for people entering the neighborhood and no information was obtained for people leaving the neighborhood (data source: surveys. The census information was not available at the level of analysis). Resulting in the characterization of a type of migratory movement that of arrival of internal migrants. We are not built other types of indicators such as net migration, gross migration, among others and focusing the analysis on the migration proportions mentioned.

### Statistical Analysis

The study sample was analyzed using relative frequencies with 95% confidence intervals (95% CI) for categorical variables and measures of central tendency and dispersion for continuous variables. Directed acyclic graphs (DAGs) guided our statistical analysis (Supplementary Figure S1). Weights were used to describe the population at the individual level. Because the probability of frailty is high, odds ratios could overestimate prevalence ratios, thus, the association of variables at the individual and contextual level with frailty was estimated using Poisson models with robust standard errors, which yields accurate prevalence ratio estimates (27). We started with an empty model (intercept only) and continued with the inclusion of individual-level variables (first level). The third model included contextual variables at the census tract level (second level). We modeled the probability of frailty using the following general equation:

$$\log\left(\lambda_{ij}\right)=\log\left(P\left(Y_{ij}=1\right)\right)=\beta_{0j}+\beta_{1}X_{ij}+\beta_{1}X_{j}+\beta_{2}Z_{j,}+\beta_{3}Z_{ij,},\beta_{0j}=\beta_{0}+{µ}_{0j}\;{µ}_{oj}∼N\;\left(0,\sigma_{\mu 0}^{2}\right)$$

Where Y_ij_ = 1 denotes the presence of frailty in person *i* in the census tract *j*. The variable X_*(ij)*_ represents the exposure variable of interest, measured as the proportion of people in urban neighborhood *j* who are migrants. The vectors Z_ij_ and Z_j_ represent covariates at the person and neighborhood level. β_oj_ is the average of log(y) (log of the prevalence of frailty) of the *j*th neighborhood.

Finally, four sensitivity analyses were conducted, we excluded the population under 18 years of age from the calculation of contextual internal migration, recognizing that adult migration is generally motivated by economic, social or individual decisions, while the migration of minors usually responds to parental decisions. The second sensitivity analysis sought to describe the behavior of the frailty prevalence ratios by categorizing the exposure variable into quartiles and whether there were differences in PR with the category originally used (Supplementary Table S1). For the third, different types of individual migration history for each participant were included as an adjustment variable to reduce the potential impact of personal migration experience on the main association (Supplementary Table S2). And fourth sensitivity analysis including forced migration (both 5 years and 1 year) as an adjustment variable in the migration models. All analyses were performed with Stata version 16.0 (StataCorp LP, College Station, United States).

## Results

The total sample consisted of 2,194 participants living in 633 census tracts from urban areas. Participants had a mean age of 68.5 years [CI 95%: 68.3, 68.7] and 53.8% of them [CI 95%: 49.0, 58.4] were women. Over half of the participants had a partner (53.3% [CI: 47.6, 58.8]), 8.6% did not have formal education [CI 95%: 5.7, 12.7], and 88.8% did not have the benefit of a retirement pension [CI 95%: 80.5, 93.8]. Prevalence of pre-frailty and frailty was 80.6% [CI 95%: 77.67, 83.28]. People with pre-frailty/frailty were in average 3 years older, more frequently women (56.9% [CI 95%: 52.1, 61.6]), without pension (91.0% [CI 95%: 87.1, 93.8]) and receiving a government support program (84.0% [CI 95%: 78.5, 88.3]) (Table 1).

**TABLE 1:** Demographic and social characteristics of study population for frailty—individual level considered sampling weights**, Colombia, 2016.

Variables	Total		No Frail		Pre & Frail	
Individual level	%	IC 95%	%	IC 95%	%	IC 95%
Prevalence			19.3	(16.7, 22.3)	80.6	(77.6, 83.2)
Age, year old (Mean)*	68.5 (68.3, 68.7)		66.7 (65.8, 67.7)		69.1 (68.8, 69.4)	
Age Cat, % *						
60–64	32.1	(27.5, 37.0)	41.1	(32.2, 50.7)	29.9	(25.7, 34.5)
65–69	30.2	(24.0, 37.3)	33.4	(25.8, 42.0)	29.5	(23.4, 36.5)
70–74	17.6	(14.4, 21.4)	12.0	(7.0, 19.9)	19.0	(16.1, 22.2)
75–79	12.5	(10.5, 14.9)	11.3	(6.3, 19.4)	12.8	(10.9, 15.0)
80–84	7.5	(6.0, 9.3)	2.1	(1.5, 3.0)	8.8	(7.0, 11.1)
Sex, % *						
Male	46.2	(41.6, 51.0)	59.4	(48.2, 69.7)	43.1	(38.4, 47.9)
Female	53.8	(49.0, 58.4)	40.6	(30.3, 51.8)	56.9	(52.1, 61.6)
Marital status, %						
Single	46.7	(41.2, 52.4)	41.4	(33.4, 50.0)	48.0	(41.6, 54.5)
Couple	53.3	(47.6, 58.8)	58.6	(50.0, 66.6)	52.0	(45.5, 58.4)
Education level, % *						
Neither	8.6	(5.7, 12.7)	5.4	(2.6, 10.6)	9.3	(6.2, 13.8)
Primary schooling	50.9	(47.6, 54.2)	35.8	(30.6, 41.4)	54.5	(50.7, 58.3)
Secondary schooling	30.6	(26.0, 35.8)	43.4	(35.6, 51.6)	27.6	(23.1, 32.6)
University education	9.2	(7.4, 11.3)	15.1	(11.4, 19.7)	7.7	(5.8, 10.2)
No data	0.7	(0.1, 3.5)	0.3	(0.0, 2.2)	0.8	(0.2, 3.9)
Wealth, % *						
Low	61.0	(54.1, 67.5)	51.5	(37.6, 65.1)	63.3	(57.7, 68.6)
Medium	37.2	(31.0, 43.9)	47.5	(34.9, 60.5)	34.7	(29.2, 40.7)
High	1.7	(0.7, 4.6)	1.0	(0.3, 3.1)	1.9	(0.7, 5.4)
Health affiliation, % *						
Insured	58.4	(51.3, 65.1)	67.6	(58.4, 75.7)	56.1	(49.3, 62.7)
Subsidized	40.8	(34.4, 47.5)	31.3	(23.7, 40.0)	43.1	(36.8, 49.7)
Uninsured	0.8	(0.4, 1.6)	1.1	(0.3, 3.5)	0.8	(0.4, 1.4)
Pension, % *						
Yes	11.2	(6.2, 19.5)	20.2	(7.2, 45.4)	9.0	(6.2, 12.9)
No	88.8	(80.5, 93.8)	79.8	(54.6, 92.8)	91.0	(87.1, 93.8)
Social programs, % *						
Yes	14.1	(10.1, 19.4)	6.3	(3.1, 12.4)	16.0	(11.7, 21.5)
No	85.9	(80.6, 89.9)	93.7	(87.6, 96.9)	84.0	(78.5, 88.3)

*p < 0.05.

**Descriptive analyses for individual’s level considered sampling weights calculated by the SABE survey.

Of the total census tracts, 62.7% (397) belonged to the very-low and low socioeconomic level, 31.7% (201) to the medium-low, and 5.5% (35) to medium, medium -high and high. Across census tracts, 70.1%, 19.6%, and 10.3% of people were in the 15 to 64, 0 to 14, and 65 or more years’ age group, respectively. For sex, women represented 52.4% of the population across census tracts. A total of 42 census tracts did not have frail adults. In the individual distribution, Table 2 shows that, of the total number of participants, 24.4% of older adults resided in neighborhoods with less than 1% of life migration in its residents, 17.0% in neighborhoods with less than 1% migrants of 5 years, and 20% with less than 1% migrants of 1 year. Of the frail participants, 43.8%, 46.9%, and 25.7% lived in neighborhoods with a percentage of lifetime, 5-year and 1-year migrants of 6% and higher.

**TABLE 2:** Distribution of the contextual migration measures among the study sample overall and by and frailty status at the individual level, Colombia, 2016.

Contextual migration	Total		No Frail		Pre & Frail	
	n	%	n	%	n	%
**Internal migration**						
lifetime						
<1%	525	24.5	98	28.9	427	23.5
1%–2%	291	13.5	46	13.7	245	13.5
3%–5%	413	19.2	68	20.1	345	19.2
6%–10%	415	19.3	51	15.1	364	20.1
11% and higher	505	23.5	75	22.2	430	23.7
All	2,149	100.0	338	100.0	1811	100.0
5-year						
<1%	363	17.2	77	22.9	286	15.9
1%–2%	298	13.9	48	14.2	250	13.9
3%–5%	501	23.4	83	24.7	418	23.2
6%–10%	602	28.2	87	25.8	515	28.6
11% and higher	370	17.3	41	12.4	329	18.4
All	2,134	100.0	336	100.0	1798	100.0
1-year						
<1%	424	19.9	85	25.3	339	18.9
1%–2%	554	26	89	26.5	465	25.9
3%–5%	631	29.5	99	29.5	532	29.5
6%–10%	396	18.6	46	13.6	350	19.5
11% and higher	129	6	17	5.1	112	6.2
All	2,134	100.0	336	100.0	1,798	100.0
**Forced migration**						
Forced 5 years						
<1%	1,277	58.2	216	62.2	1,061	57.4
1%–2%	675	30.8	99	28.5	576	31.2
3% and higher	242	11.0	32	9.2	210	11.4
All	2,194	100.0	347	100.0	1,847	100.0
Forced 1 year						
<1%	1,723	80.7	282	83.9	1,441	80.1
1%–2%	356	16.7	46	13.7	310	17.2
3% and higher	55	2.6	8	2.4	47	2.6
All	2,134	100.0	336	100.0	1,798	100.0

**FIGURE 1:**
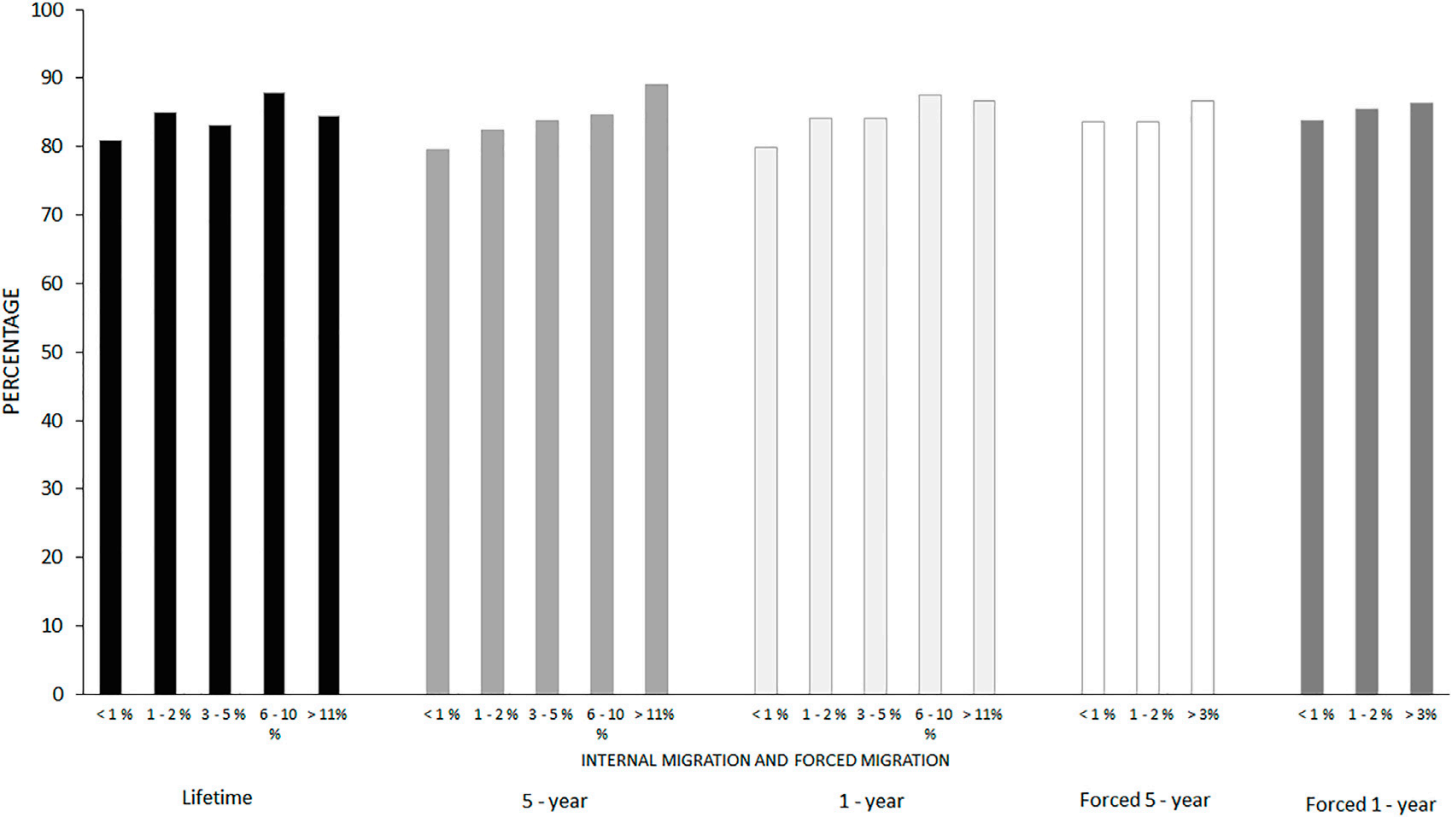
Prevalence of pre/frailty by type of migration and percentage of migrants in the neighborhood, Colombia, 2016.

Figure 1 shows the prevalence of pre/frailty among older adults in neighborhoods with different levels of internal contextual migration. In neighborhoods with less than 1% migration, the prevalence of pre/frailty was up to 80% for all types of migration. The prevalence of pre/frailty increased as the percentage of migrants increased, being higher than 84% in neighborhoods with 11% or more migrants. For forced migration, the differences were not as evident as for internal contextual migration in general, but an increasing trend is observed as the percentage of migrants in the neighborhood increases.

Table 3 shows the results of the analysis of the association of all types of contextual internal migration with pre-frailty and frailty. The prevalence ratio of pre-frailty/frailty increases as the proportion of the migrant population in the neighborhood increases, being greater and statistically significant for participants who lived in neighborhoods with more than 6% of the migrant population in their environment. In the crude model, the prevalence ratio of pre-frailty/frailty in older adults was 1.07 (CI 95%: 1.01, 1.14), comparing neighborhoods with 6% or more contextual lifetime migrants to neighborhoods with <1% lifetime migrants. This association remained when adjusting for all the covariates considered in model 3 (PR = 1.08, CI 95%: 1.02, 1.15).

**TABLE 3:** Multilevel model results for pre/frailty and internal and forced contextual migration categories, Colombia, 2016.

Contextual migration	Model 1		Model 2		Full model	
	PR	95% IC	PR	95% IC	PR	95% IC
Lifetime						
<1%	Ref		Ref		Ref	
1%–2%	1.03	(0.95, 1.12)	1.04	(0.96, 1.12)	1.05	(0.97, 1.13)
3%–5%	1.02	(0.96, 1.09)	1.02	(0.96, 1.09)	1.02	(0.96, 1.09)
6%–10%	**1.07**	(1.01, 1.14)	**1.08**	(1.01, 1.14)	**1.08**	(1.02, 1.15)
11% and higher	1.04	(0.98, 1.11)	**1.04**	(0.99, 1.10)	1.04	(0.99, 1.10)
5-year						
<1%	Ref		Ref		Ref	
1%–2%	1.06	(0.98, 1.15)	1.03	(0.95, 1.11)	1.03	(0.95, 1.12)
3%–5%	1.05	(0.98, 1.14)	1.04	(0.97, 1.12)	1.05	(0.97, 1.13)
6%–10%	**1.08**	(1.00, 1.16)	1.05	(0.98, 1.13)	1.06	(0.99, 1.14)
11% and higher	**1.12**	(1.04, 1.21)	**1.11**	(1.03, 1.20)	**1.11**	(1.03, 1.20)
1-year						
<1%	Ref		Ref		Ref	
1%–2%	1.04	(0.98, 1.12)	1.04	(0.97, 1.12)	1.05	(0.98, 1.12)
3%–5%	1.05	(0.98, 1.12)	1.05	(0.98, 1.12)	1.05	(0.98, 1.12)
6%–10%	**1.10**	(1.03, 1.18)	**1.09**	(1.02, 1.16)	**1.09**	(1.02, 1.16)
11% and higher	**1.08**	(1.00, 1.18)	**1.08**	(1.00, 1.18)	**1.08**	(1.00, 1.18)

Model 1: Frailty and migration. Model 2: Adjusted for sex. age. education. SES. Pension. Health Affiliation. social programs. living arrangements (couple/brothers/friends), individual experience of migration. Model 3: Adjusted for sex. age. education. SES. Pension. Health Affiliation. Living arrangements + SES neighborhood and percentage of 15–64 in the neighborhood.

Bold values: *p* < 0.05.

Although attenuated, the association between contextual internal and frailty remained statistically significant when adjusting for all the covariates when comparing neighborhoods with 11% and higher versus those with <1% of 5-year migrants (model 3, PR = 1.11, CI 95%: 1.03, 1.20). For contextual internal migration in the last year, the prevalence ratios were similar across models, being statistically significant for neighborhoods with 6%–10% and 11% and higher versus <1% of migrants (model 3, PR: 1.09, CI95% 1.02, 1.16 for the category of 6%–10%; PR: 1.08, CI 95%: 1.00, 1.18 for the category of 11% and higher).

Forced migration estimators followed a similar pattern to internal migration. The prevalence of pre-frailty/frailty increased as the percentage of forced migrants in the neighborhood increased, although associations were not statistically significant (Table 4).

**TABLE 4:** Multilevel model results for pre/frailty and Neighborhood forced migrants, Colombia, 2016.

Contextual migration	Forced 5-year		Forced 1-year	
	PR	95%CI	PR	95%CI
<1%	Ref		Ref	
1%–2%	0.99	(0.95, 1.04)	1.02	(0.96, 1.08)
3% and higher	1.03	(0.98, 1.10)	1.03	(0.93, 1.15)

Model 1: Frailty and forced migration. Model 2: Adjusted for sex. age. education. SES. Pension. Health Affiliation. social programs. living arrangements (couple/brothers/friends), individual experience of migration. Model 3: Adjusted for sex. age. education. SES. Pension. Health Affiliation. Living arrangements + SES neighborhood and percentage of 15–64 in the neighborhood.

### Sensitivity Analysis

**FIGURE 2:**
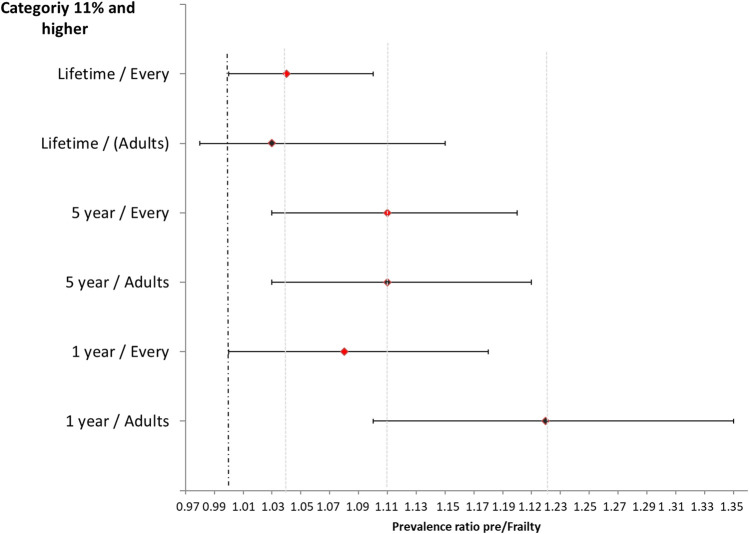
Prevalence ratio behavior for frailty in relation to types internal migration (>6%) for the entire population and only adults, Colombia, 2016.

The estimated associations of the three types of contextual internal migration with pre/fragility excluding the population under 18 years of age for lifetime migrants had the same direction as the main results, but were not statistically significant. For 5-year migration, results were similar to those obtained when considering the entire population, and for migration of 1 year, the prevalence ratio increased from 1.08 to 1.22 (Figure 2). In the second sensitivity analysis, the estimations obtained by categorizing the different types of migration in quartiles (Supplementary Table S1) remained similar to those observed when classifying by percentages. The prevalence ratio of pre-frailty/frailty increased as the proportion of the migrant population in the neighborhood increased, being higher and statistically significant for older adults who lived in neighborhoods within the fourth quartile of the migrant population. For the third sensitivity analysis, the estimated associations of the three types of contextual internal migration with pre/frailty when including the migratory background of the elderly in the final models were similar to those obtained when considering the entire population (Supplementary Table S2). And the fourth analysis when including forced migration (both 5 years and 1 year) as an adjustment variable in the migration models, no major changes were observed (Supplementary Table S3). Only a slight increase was observed in the prevalence of pre-frailty/frailty in neighborhoods with internal migration 11% and higher versus <1% within the past 5 years (PR = 1.12, CI 95%: 1.03, 1.21).

## Discussion

In this study, we aimed to analyze the association between contextual levels of internal and forced migration at the neighborhood level and frailty among older adults in Colombia. Most previous research has focused instead on individual experiences of migration instead of contextual effects. We found that the prevalence of pre-frailty/frailty was significantly higher in the elderly living in neighborhoods with a high proportion of internal migration compared to participants living in neighborhoods with low internal migration. We did not observe the same behavior for forced migration, where the proportion of forced migration in the neighborhood did not show an association with frailty.

The influence of contextual internal and forced migration on health outcomes has been scarcely studied. Specifically, to our knowledge no studies about the impacts of contextual migration on frailty are available, precluding the comparison of our findings with similar studies. Studies about frailty and migration are based on the migration background history of the elderly especially with international migration, where a greater probability of frailty has been evidenced in migrants from low-income countries based on ethnic origin (29, 30), the ethnic density of the neighborhood (living in a neighborhood characterized by a higher density of African Americans and with more residential instability) (31), the loss of protective social networks with experiences of discrimination and language barriers (19, 32) and in early adverse experiences that are reflected in old age (20, 29). One study did not show this association between a history of migration and frailty (33).

In Colombia, we observed that contextual internal migration was positively associated with frailty. Several potential mechanisms could explain this association, related to how internal migration could change the neighborhood environment or its perception from the perspective of older adults. According to social stress theory, if internal migration is negatively perceived, receiving migrants in the neighborhood could constitute a stressor (16), activating neuroinflammatory signaling that provokes inflammatory mediators (interleukins/cortisol), predisposing older adults to frailty (6, 15). In this way, internal migration could affect the perception or the objective conditions of the social environment, the physical and built environment and become a stressor for older adults. As seen with the arrival of refugees in a community in Ethiopia, the socio-cultural norms of the host peoples were altered in terms of their social insecurity and the introduction of health-related challenges (34).

From human-ecological theory (35) and social environment perspective, the increase in the proportion of internal migrants in the neighborhood increases cultural heterogeneity (28), which may be related to an increase in the perception of insecurity and the rejection of migrants by long-term inhabitants (36), with feelings of resentment that prevent social integration and the inclusion of the host population in the process of adaptation of migrants or refugees (37). Residents could also perceive migrants as a threat to the social structure and resent the external aid received by migrants (38). Other aspect are when the older adults are be considered the minority group the neighborhood leading them to be potentially discriminated against because of their age and your health factors being affected (28, 39, 40).

From the perspective of physical environments, the changes in the perception of neighborhood cohesion and safety show other mechanisms that could directly impact frailty through changes in the physical environment. This is especially relevant for frailty criteria of physical activity and prehensile strength (21) as the arrival of Internal migrants can put pressure on physical environments that were used by older adults, potentially reducing their interactions with the built environment and therefor reducing utilitarian or leisure walking or by reducing the attendance to recreational facilities subsequently decreasing physical activity in the elderly (41).

While we found that internal migration was associated with increased frailty in older adults, this association is likely to vary according to the characteristics of the receiving community (17, 18) and the intensity and characteristics of the migration process. Rapid migration of large groups, such as the one frequently experienced in Colombia, will likely produce a larger disruption in the receiving community due to perceived threats to identity, employment, and socio-cultural and religious values (36). Also, the characteristics of the receiving community could modify the effect of migration; for instance, if the community has a strong economic and social structure that facilitates the integration of migrants, that could reduce the negative perception of internal migration (17, 18).

Limitations include the cross-sectional design that precludes a causal interpretation of the relationship between internal migration and pre/frailty. Also, we do not rule out residual confusion due to variables not considered in the study or the analysis; for example, the density of local resources in the neighborhood can show the strengths or weaknesses of the neighborhood to welcome the migrant population, or the history of migration in the neighborhood. Also frailty is a complex construct linked to many factors so it’s hard to argue that migration itself impacts frailty in a direct way. There are many other factors related to frailty that could co vary with migration, also lifecourse processes.

Not being able to characterize the different migratory flows at the neighborhood level (includes exit and entry) could lead to implications such as not being able to identify attractive or rejection neighborhoods for migrants and with them the possibility of relating them to characteristics of older adults for the benefit or not of the frailty development. And another relevant aspect is not accurately knowing the spatial redistribution of the population in the period studied based on migratory flows, which would allow to some extent to know social, cultural and economic effects in the environment of the receiving and sending place of migrants and with this, quality of life variables that can influence the wellbeing of the older adults.

We found that internal migration is associated with frailty among the elderly. It is important to recognize the health status of the populations that host migrants, evaluating local resources. The internal migratory processes in the country should not be stigmatized, considering migrants as actors of cohesion and social development of the neighborhoods from the construction from the differences. We seek to fill the knowledge gap around the social etiology of frailty outside the individual context of the elderly. We also highlight the importance of studying internal migration in a country with social characteristics such as Colombia and in the urban environment.
