# Supplementary material for: Frailty in Older Adults and Internal and Forced Migration in Urban Neighborhood Contexts in Colombia
Source: Int J Public Health. 2023 May 5;68:1605379. doi: 10.3389/ijph.2023.1605379 (PMC10196000; doi:10.3389/ijph.2023.1605379)
Supplement: Supplementary file 1 [file DataSheet1.zip › Suplementary/Figure S1.docx]

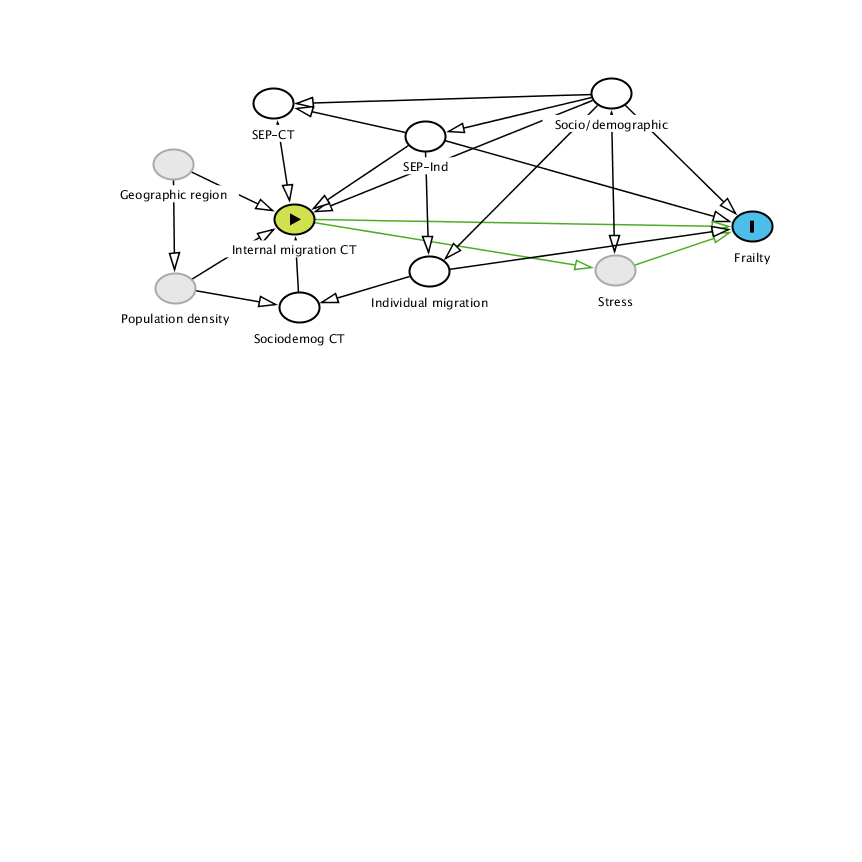


Figure S1. Directed acyclic graphs (DAGs) Frailty in older adults and internal and forced migration in urban neighborhood contexts

**Socio/demographic**: sex, age, educational level, marital status, Living arrangements, health insurance, pension (yes/no). **SEP-ind**: Socioeconomic position-individual level. **Individual migration**: individual experience of migration adults older. **Internal migration CT**: contextual internal migration – census tract level / contextual forced migration. **SEP-CT**: socioeconomic level at the census tract level. **Socio/demographic**: age distribution (proportion of people 0 to 14, 15 to 64, and 65 and over). **Geographic region:** geographic region. **Population density**: population density. **Physical CT**: neighborhood built environment. **Social CT**: social environment of the neighborhood, social cohesion, perception of insecurity, violence, etc.
