# Supplementary material for: Frailty in Older Adults and Internal and Forced Migration in Urban Neighborhood Contexts in Colombia
Source: Int J Public Health. 2023 May 5;68:1605379. doi: 10.3389/ijph.2023.1605379 (PMC10196000; doi:10.3389/ijph.2023.1605379)
Supplement: Supplementary file 1 [file DataSheet1.zip › Suplementary/Table S2.docx]

Table S2. Multilevel model results for frailty and Neighborhood migration with the categorization of contextual migration by quartiles, Colombia, 2016.

| **Contextual migration** | **Lifetime** | | **5-year** | | **1-year** | |
| --- | --- | --- | --- | --- | --- | --- |
|  | **PR** | **95%CI** | **PR** | **95%CI** | **PR** | **95%CI** |
| 1 Q | Ref | | Ref | | Ref | |
| 2 Q | 1.05 | (0.99, 1.11) | 1.02 | (0.97, 1.08) | **1.07** | (1.01, 1.12) |
| 3 Q | **1.06** | (1.00, 1.12) | 1.03 | (0.97, 1.09) | 1.04 | (0.98, 1.10) |
| 4 Q | **1.07** | (1.01, 1.12) | **1.09** | (1.03, 1.14) | **1.08** | (1.02, 1.14) |

Model 1: Frailty and migration. Model 2: Adjusted for sex. age. education. SES. Pension. Health Affiliation. social programs. living arrangements. Model 3: Adjusted for sex. age. education. SES. Pension. Health Affiliation. Living arrangements + SES neighborhood and population of 15 to 64 in the neighborhood.
