# Supplementary material for: Frailty in Older Adults and Internal and Forced Migration in Urban Neighborhood Contexts in Colombia
Source: Int J Public Health. 2023 May 5;68:1605379. doi: 10.3389/ijph.2023.1605379 (PMC10196000; doi:10.3389/ijph.2023.1605379)
Supplement: Supplementary file 1 [file DataSheet1.zip › Suplementary/Table S3.docx]

|  | **Migration history of the older adults** | | | | | | | | | |
| --- | --- | --- | --- | --- | --- | --- | --- | --- | --- | --- |
| **Contextual internal** | **5 - year migrant** | | **Lifetime forced migration** | | **5 - year forced migration** | | **Family migration** | | **Individual migration** | |
|  | **PR** | **95% IC** | **PR** | **95% IC** | **PR** | **95% IC** | **PR** | **95% IC** | **PR** | **95% IC** |
| **Lifetime** |  |  |  |  |  |  |  |  |  |  |
| < 1 % | Ref | | Ref | | Ref | | Ref | |  | |
| 1 - 2 % | 1.05 | (0.97, 1.13) | 1.05 | (0.97, 1.13) | 1.05 | (0.97, 1.13) | 1.05 | (0.97, 1.13) | 1.05 | (0.97, 1.13) |
| 3 - 5 % | 1.02 | (0.96, 1.09) | 1.02 | (0.96, 1.09) | 1.02 | (0.96, 1.09) | 1.02 | (0.96, 1.09) | 1.02 | (0.96, 1.09) |
| 6 - 10 % | **1.08** | (1.02, 1.15) | **1.08** | (1.02, 1.15) | **1.08** | (1.02, 1.15) | **1.09** | (1.02, 1.15) | **1.08** | (1.02, 1.15) |
| 11% and higher | 1.04 | (0.98, 1.10) | 1.04 | (0.98, 1.10) | 1.04 | (0.98, 1.10) | 1.04 | (0.99, 1.10) | 1.04 | (0.98, 1.10) |
| **5 - year** | | |  |  |  |  |  |  |  |  |
| < 1 % | Ref | | Ref |  | Ref |  | Ref |  | Ref |  |
| 1 - 2 % | 1.03 | (0.95, 1.12) | 1.03 | (0.95, 1.12) | 1.03 | (0.95, 1.12) | 1.03 | (0.95, 1.12) | 1.03 | (0.95, 1.12) |
| 3 - 5 % | 1.05 | (0.97, 1.13) | 1.05 | (0.97, 1.13) | 1.05 | (0.97, 1.13) | 1.05 | (0.97, 1.13) | 1.05 | (0.97, 1.13) |
| 6 - 10 % | 1.06 | (0.98, 1.14) | 1.06 | (0.98, 1.14) | 1.06 | (0.98, 1.14) | 1.06 | (0.99, 1.14) | 1.06 | (0.98, 1.14) |
| 11% and higher | **1.11** | (1.03, 1.20) | **1.11** | (1.03, 1.20) | **1.11** | (1.03, 1.20) | **1.11** | (1.03, 1.20) | **1.11** | (1.03, 1.20) |
| **1 - year** |  |  |  |  |  |  |  |  |  |  |
| < 1 % | Ref |  | Ref |  | Ref |  | Ref |  | Ref |  |
| 1 - 2 % | 1.05 | (0.98, 1.12) | 1.05 | (0.98, 1.12) | 1.05 | (0.98, 1.12) | 1.05 | (0.98, 1.12) | 1.05 | (0.98, 1.12) |
| 3 - 5 % | 1.05 | (0.98, 1.12) | 1.05 | (0.98, 1.12) | 1.05 | (0.98, 1.12) | 1.05 | (0.98, 1.12) | 1.05 | (0.98, 1.12) |
| 6 - 10 % | **1.09** | (1.02, 1.16) | **1.09** | (1.02, 1.16) | **1.09** | (1.02, 1.16) | **1.09** | (1.02, 1.16) | **1.09** | (1.02, 1.16) |
| 11% and higher | **1.08** | (1.00, 1.18) | **1.08** | (1.00, 1.18) | **1.08** | (1.00, 1.17) | **1.08** | (1.00, 1.17) | **1.08** | (1.00, 1.17) |

**Table S3**. Multilevel models for the comparison of the frailty prevalence ratio, including adjustment for the migratory history of older adults in its different forms (5-year-old migrant, forced lifetime migration, 5-year forced migration, family migration, individual)
