# Supplementary material for: Frailty in Older Adults and Internal and Forced Migration in Urban Neighborhood Contexts in Colombia
Source: Int J Public Health. 2023 May 5;68:1605379. doi: 10.3389/ijph.2023.1605379 (PMC10196000; doi:10.3389/ijph.2023.1605379)
Supplement: Supplementary file 1 [file DataSheet1.zip › Suplementary/Table S1.docx]

Table S1-1. Distribution of the contextual variables and frailty status at the neighbourhood level, Colombia, 2016.

| Contextual variables (Neighbourhood level) | No Frail |  | Frailty |  | Total |  |
| --- | --- | --- | --- | --- | --- | --- |
|  | **n** | **%** | **n** | **%** | **n** | **%** |
| Socioeconomic level that is most repeated in the neighborhood* | | |  |  |  |  |
| Level 1 (SEL-1) | 80 | 23.05% | 521 | 28.21% | 601 | 27.39% |
| Level 2 (SEL-2) | 149 | 42.94% | 816 | 44.18% | 965 | 43.98% |
| Level 3 (SEL-3) | 102 | 29.39% | 454 | 24.58% | 556 | 25.34% |
| Level 4 (SEL-4) | 10 | 2.88% | 33 | 1.79% | 43 | 1.96% |
| Level 5 (SEL-5) | 6 | 1.73% | 9 | 0.49% | 15 | 0.68% |
| Level 6 (SEL-6) | 0 | 0.00% | 14 | 0.76% | 14 | 0.64% |
| Proportion of men in neighborhood |  |  |  |  |  |  |
| 40 - 44% | 17 | 4.90% | 54 | 2.92% | 71 | 3.24% |
| 45 - 49% | 310 | 89.34% | 1683 | 91.12% | 1993 | 90.84% |
| 50 - 54% | 20 | 5.76% | 110 | 5.96% | 130 | 5.93% |
| Greater than 50% |  |  |  |  |  |  |
| Proportion of population from 0 to 14 years | |  |  |  |  |  |
| 1 - 19% | 173 | 49.86% | 864 | 46.78% | 1037 | 47.27% |
| 20 - 39% | 174 | 50.14% | 983 | 53.22% | 1157 | 52.73% |
| Proportion of population from 15 to 64 years** | |  |  |  |  |  |
| 40 - 59% | 2 | 0.58% | 10 | 0.54% | 12 | 0.55% |
| 60 - 79% | 342 | 98.56% | 1834 | 99.30% | 2176 | 99.18% |
| 80 - 100% | 3 | 0.86% | 3 | 0.16% | 6 | 0.27% |
| Proportion of population older than 65 years | |  |  |  |  |  |
| 1 - 19% | 343 | 98.85% | 1818 | 98.43% | 2161 | 98.50% |
| 20 - 39% | 4 | 1.15% | 29 | 1.57% | 33 | 1.50% |
| Total | 347 | 100.00% | 1847 | 100.00% | 2194 | 100.00% |

*p: < 0.05

** p: 0.05 – 0.10

Table S1-2. Distribution of the contextual variables and internal migration status at the neighbourhood level, Colombia, 2016.

| Contextual variables (Neighbourhood level) | **Internal migration** | | | | | | | | | | | | | | |
| --- | --- | --- | --- | --- | --- | --- | --- | --- | --- | --- | --- | --- | --- | --- | --- |
|  | lifetime | | | | | 5 - year |  |  |  |  | 1 - year |  |  |  |  |
|  | Not | | Yes | | Total | Not | | Yes | | Total | Not |  | Yes |  | Total |
|  | n | % | n | % | n | n | % | n | % | n | n | % | n | % | n |
| **Socioeconomic level that is most repeated in the neighborhood*** | | | | | | | | | | | | | | | |
| Level 1 (SEL-1) | 113 | 29.3 | 273 | 70.7 | 386 | 64 | 16.7 | 318 | 83.2 | 382 | 75 | 19.6 | 307 | 80.3 | 382 |
| Level 3 (SEL-3) | 62 | 31.9 | 132 | 68.0 | 194 | 44 | 23.1 | 146 | 76.8 | 190 | 60 | 31.5 | 130 | 68.4 | 190 |
| Level 4 (SEL-4) | 12 | 50.0 | 12 | 50.0 | 24 | 9 | 37.5 | 15 | 62.5 | 24 | 14 | 58.3 | 10 | 41.6 | 24 |
| Level 5 (SEL-5) | 2 | 28.5 | 5 | 71.4 | 7 | 4 | 66.6 | 2 | 33.3 | 6 | 2 | 33.3 | 4 | 66.6 | 6 |
| Level 6 (SEL-6) | 1 | 25.0 | 3 | 75.0 | 4 | 2 | 50.0 | 2 | 50.0 | 4 | 1 | 25.0 | 3 | 75.0 | 4 |
| **Proportion of men in neighborhood** | | | | | | | | | | | | | | | |
| 40 - 44% | 15 | 44.1 | 19 | 55.8 | 34 | 11 | 31.4 | 24 | 68.5 | 35 | 12 | 34.2 | 23 | 65.7 | 35 |
| 45 - 49% | 168 | 30.4 | 384 | 69.5 | 552 | 108 | 20.0 | 432 | 80.0 | 540 | 134 | 24.8 | 406 | 75.1 | 540 |
| 50 - 54% | 7 | 24.1 | 22 | 75.8 | 29 | 4 | 12.9 | 27 | 87.0 | 31 | 6 | 19.3 | 25 | 80.6 | 31 |
| **Proportion of population from 0 to 14 years** | | ** | | | | * | | | | | * | | | | |
| 1 - 19% | 112 | 33.2 | 225 | 66.7 | 337 | 83 | 25.3 | 244 | 74.6 | 327 | 104 | 31.8 | 223 | 68.1 | 327 |
| 20 - 39% | 78 | 28.0 | 200 | 71.9 | 278 | 40 | 14.3 | 239 | 85.6 | 279 | 48 | 17.0 | 231 | 82.7 | 279 |
| **Proportion of population from 15 to 64 years** | | | | | | | | | | | * | | | | |
| 40 - 59% | 1 | 50.0 | 1 | 50.0 | 2 | 0 | 0.0 | 2 | 100.0 | 2 | 0 | 0.0 | 2 | 100.0 | 2 |
| 60 - 79% | 189 | 30.9 | 422 | 69.0 | 611 | 123 | 20.3 | 480 | 79.6 | 603 | 152 | 25.2 | 451 | 74.7 | 603 |
| 80 - 100% | 0 | 0 | 2 | 100 | 2 | 0 | 0.0 | 1 | 100 | 1 | 0 | 0.0 | 1 | 100 | 1 |
| **Proportion of population older than 65 years** | | * | | | | * | | | | |  | | | | |
| 1 - 19% | 182 | 30.2 | 420 | 69.7 | 602 | 115 | 19.3 | 478 | 80.6 | 593 | 144 | 24.2 | 449 | 75.7 | 593 |
| 20 - 39% | 8 | 61.5 | 5 | 38.4 | 13 | 8 | 61.5 | 5 | 38.4 | 13 | 8 | 61.5 | 5 | 38.4 | 13 |
| Total | 190 | 30.8 | 425 | 69.1 | 615 | 123 | 20.3 | 483 | 79.7 | 606 | 152 | 25.0 | 454 | 74.9 | 606 |

*p: < 0.05

** p: 0.05 – 0.10
